# Differentiation States of Phenotypic Transition of Melanoma Cells Are Revealed by 3D Cell Cultures

**DOI:** 10.3390/cells13020181

**Published:** 2024-01-17

**Authors:** Fabrizio Fontana, Michele Sommariva, Martina Anselmi, Francesca Bianchi, Patrizia Limonta, Nicoletta Gagliano

**Affiliations:** 1 Department of Pharmacological and Biomolecular Sciences “Rodolfo Paoletti”, Università degli Studi di Milano, 20133 Milan, Italy; fabrizio.fontana@unimi.it (F.F.); martina.anselmi@unimi.it (M.A.); patrizia.limonta@unimi.it (P.L.); 2 Department of Biomedical Sciences for Health, Università degli Studi di Milano, 20133 Milan, Italy; michele.sommariva@unimi.it (M.S.); francesca.bianchi1@unimi.it (F.B.); 3 U. O. Laboratorio Morfologia Umana Applicata, IRCCS Policlinico San Donato, San Donato Milanese, 20097 Milan, Italy

**Keywords:** melanoma, 3D spheroids, epithelial-to-mesenchymal transition, E-cadherin, MMPs, LOX

## Abstract

Melanoma is characterized by high metastatic potential favored by the epithelial-to-mesenchymal transition (EMT), leading melanoma cells to exhibit a spectrum of typical EMT markers. This study aimed to analyze the expression of EMT markers in A375 and BLM melanoma cell lines cultured in 2D monolayers and 3D spheroids using morphological and molecular methods. The expression of EMT markers was strongly affected by 3D arrangement and revealed a hybrid phenotype for the two cell lines. Indeed, although E-cadherin was almost undetectable in both A375 and BLM cells, cortical actin was detected in A375 2D monolayers and 3D spheroids and was strongly expressed in BLM 3D spheroids. The mesenchymal marker N-cadherin was significantly up-regulated in A375 3D spheroids while undetectable in BLM cells, but vimentin was similarly expressed in both cell lines at the gene and protein levels. This pattern suggests that A375 cells exhibit a more undifferentiated/mesenchymal phenotype, while BLM cells have more melanocytic/differentiated characteristics. Accordingly, the Zeb1 and 2, Slug, Snail and Twist gene expression analyses showed that they were differentially expressed in 2D monolayers compared to 3D spheroids, supporting this view. Furthermore, A375 cells are characterized by a greater invasive potential, strongly influenced by 3D arrangement, compared to the BLM cell line, as evaluated by SDS-zymography and TIMPs gene expression analysis. Finally, TGF-β1, a master controller of EMT, and lysyl oxidase (LOX), involved in melanoma progression, were strongly up-regulated by 3D arrangement in the metastatic BLM cells alone, likely playing a role in the metastatic phases of melanoma progression. Overall, these findings suggest that A375 and BLM cells possess a hybrid/intermediate phenotype in relation to the expression of EMT markers. The former is characterized by a more mesenchymal/undifferentiated phenotype, while the latter shows a more melanocytic/differentiated phenotype. Our results contribute to the characterization of the role of EMT in melanoma cells and confirm that a 3D cell culture model could provide deeper insight into our understanding of the biology of melanoma.

## 1. Introduction

Cutaneous malignant melanoma is a skin cancer originating not only from highly proliferating differentiated adult melanocytes, but also from melanocyte stem cells (MSCs). Although it accounts for only 5% of cutaneous tumors, it still represents the cause of about 75% of skin cancer-related deaths [1]. The five-year survival rate is higher than 90% for the early stages, supporting the acquisition of a metastatic phenotype [2,3].

The development and progression of melanoma includes two phases: the radial and the vertical growth phase. In the radial phase (melanoma in situ), neoplastic melanocytes spread radially within the basal epidermis. In the vertical phase (tumorigenic melanoma), transformed cells grow vertically, degrading the basement membrane and invading deeper tissues, thus becoming metastatic [4].

Similar to carcinomas, melanoma’s progression is a complex process including different steps, such as invasion of the adjacent tissue, transendothelial migration into blood vessels, survival in the circulation, extravasation and colonization of secondary tumor sites.

In this progression, the “epithelial-to-mesenchymal transition” (EMT) leads epithelial cancer cells to lose their typical histologic features and to acquire a mesenchymal-like phenotype able to support their migratory and invasive potential [5,6]. EMT transition is characterized by the loss of epithelial markers, such as E-cadherin, claudins, and cytokeratins, and the up-regulation of mesenchymal markers, such as N-cadherin, vimentin, fibronectin, and alpha-smooth muscle actin (αSMA) [5,7,8,9].

Although melanoma cells are not of epithelial origin, they exhibit a spectrum of typical EMT markers and undergo an EMT-like process similar to that observed in carcinomas [10]. This includes the so-called “cadherin switch” characterized by E-cadherin down-regulation and N-cadherin expression [11]. Interestingly, this EMT-like process is driven by complex transcriptional pathways that mimic the formation and migration of melanocytes from the neural crest to the epidermis during embryogenesis [4,12,13,14].

The EMT molecular events are finely orchestrated by several transcription factors, such as Twist, Zeb, Snail/SNAI1 and Slug/SNAI2, which play key roles especially in E-cadherin down-regulation and the acquisition of the mesenchymal phenotype [9,15,16,17]. The overall EMT process is influenced by transforming growth factor-β1 (TGF-β1), which is also able to control tumor cell motility and invasive behavior [18,19].

Although, during EMT, a complete conversion of epithelial into mesenchymal cells can occur, cancer cells often undergo an incomplete or partial EMT, resulting in the acquisition of a hybrid phenotype with the concomitant expression of both epithelial and mesenchymal markers [7,8]. Similar to the epithelial context, melanomas are also associated with different cellular phenotypes, leading to high tumor heterogeneity. Indeed, up to seven different melanoma phenotypic states have been described so far [20,21,22], characterized by rapid proliferation and lower invasion (proliferative or melanocytic state) or by slow proliferation and high invasion (invasive/mesenchymal-like or undifferentiated state) [10,23]. The epithelial—hybrid—mesenchymal transition develops on a vertical phenotypic gradient from the upper to the deeper part of invasive melanoma. Cells exhibiting melanocytic/differentiated states are more superficial, while less differentiated/mesenchymal cells are located deep in the invasive front of the tumor [24].

The expression of matrix metalloproteinases (MMPs) and their inhibitors (TIMPs) also plays a key role during melanoma’s progression, contributing to the extracellular matrix (ECM) remodeling needed for tumor invasion, migration, and metastasis. MMP-2 and MMP-9 are overexpressed in melanoma cells and positively correlated with highly metastatic tumor behavior [4], while their endogenous inhibitors, TIMP-1 and -2, are down-regulated in melanoma cells, exhibiting aggressive potential [25,26]. In line with this, the TIMP/MMP axis is now considered a promising molecular target for the treatment of melanoma patients [27].

Recent evidence also highlights the role of lysyl oxidase (LOX) and the LOX family members in melanoma’s progression and metastasis for their involvement in collagen maturation, and, therefore, for their influence on the ability of cancer cells to remodel the ECM during the invasion process [28,29,30].

More recently, a growing body of evidence has demonstrated that 3D culture systems better recapitulate the complex biological and molecular features of tumor tissues by mimicking the tumor architecture, including cell—cell and cell—microenvironment interactions. The 3D cell cultures also contribute to achieving a better insight into the mechanisms responsible for therapeutic escape and drug resistance, representing an effective tool to bridge the gap between the 2D in vitro and in vivo experimental models [31,32,33]. So far, advantages of the 3D cell systems over the “classical” 2D cultures have been reported for different cancer cells, including melanoma [34,35,36,37,38,39]. These studies have generated information about the metabolism and the response to drugs of melanoma cells, but a detailed characterization of EMT-related pathways in melanoma 3D cell cultures is still lacking.

In this study, we aimed to analyze the effect of the 3D arrangement on the morphological features and expression of some peculiar key EMT markers in two different melanoma cells lines: A375 (BRAF V600E-mutant, the predominant BRAF mutation occurring in about 50% of cases), isolated from a primary melanoma, and BLM (NRAS-mutant, a mutation present in about 30% of patients), isolated from a lung metastasis. The mutant NRAS protein constitutively activates the mitogen activated protein kinase (MAPK), phosphoinositide 3-kinase (PI3K)/AKT/mTOR, and Ral pathways, which favor uncontrolled cell proliferation and survival, eventually influencing the tumor’s clinical and prognostic behavior as well as its sensitivity to therapies [40].

Our results show that both cell lines possess a hybrid phenotype with regard to the expression of EMT markers exhibiting intermediate phenotypic states. These results support the advantage of the application of 3D spheroid culture techniques in the understanding of melanoma biology.

## 2. Materials and Methods

### 2.1. Cell Cultures

The human melanoma cancer cell line A375 was purchased from American Type Culture Collection (ATCC, Manassas, VA, USA). The BLM human melanoma cell line was kindly provided by Dr. G.N. van Muijen from Radboud University Nijmegen Medical Center (Department of Pathology, Nijmegen, The Netherlands). Cells were cultured at 37 °C in a humidified atmosphere containing 5% CO_2_ in Dulbecco’s modified eagle’s medium (DMEM) and 10% heat-inactivated fetal bovine serum (FBS), 2 mM glutamine, antibiotics (100 U/mL penicillin, 0.1 mg/mL streptomycin), and 0.25 μg/mL amphotericin B. Cells were cultured in T25 flasks, and their viability was determined using Trypan blue staining.

Melanoma 2Dmonolayers were cultured in duplicate for each cell line (two copies of the same sample) in T25 flasks (Euroclone, Pero, Milan, Italy). Melanoma 3D spheroids were obtained in low-attachment conditions after seeding cells (5 × 10^3^ cells) in 1% agarose coated 24-well plates (1 spheroid in each well). Spheroid formation was evident after 3 days, and integrity was verified by phase-contrast microscopy. According to the protocol standardized in our laboratory [36], spheroids were harvested 7 days after seeding for morphological and molecular evaluations before. Then, 3D spheroids cultured in 24-well plates were pooled to obtain duplicate samples for each cell line (for each duplicate sample approximately 120 spheroids were pooled to obtain two independent samples for each cell line). Biological duplicates were analyzed separately.

### 2.2. Real Time PCR

After harvesting melanoma 2D monolayers and 3D spheroids, total RNA was extracted (Tri-Reagent, Sigma-Aldrich, Milan, Italy) and reverse-transcribed (Biorad, Segrate, Milan, Italy). The gene expression of E-cadherin, N-cadherin, vimentin, Zeb1, Zeb2, Twist, Snail, Slug, TIMP-1, TIMP-2, TGF-β1 and LOX was analyzed by real-time RT-PCR. Two independent copies of each sample were analyzed. Each sample was run in triplicate (each sample was loaded in three wells of a 96-well PCR plate) in each amplification, and each amplification was repeated three times in 96-well plates. Glyceraldehyde 3-phosphate dehydrogenase (GAPDH) served as the housekeeping gene for data normalization. The primers sequences were the following: GAPDH: forward CCCTTCATTGACCTCAACTACATG, reverse TGGGATTTCCATTGATGACAAGC; E-cadherin: forward GAACGCATTGCCACATACAC; GAATTCGGGCTTGTTGTCAT; N-cadherin: forward TGTTTGACTATGAAGGCAGTGG, reverse TCAGTCATCACCTCCACCAT; vimentin forward TGGTCCTACCCACGCAGATT, reverse GGCCAACCCAGAAGTTGGAA; Zeb1: forward GCCAATAAGCAAACGATTCTG; reverse TTTGGCTGGATCACTTTCAAG; Zeb2: forward GCTACACGTTTTGCCTACCGC; reverse CGATTACCTGCTCCTTTGGGTT; Twist: forward TGAGCAAGATTCAGACCCTCA, reverse ATCCTCCAGACCGAGAAGG; Snail: forward CTTCCAGCAGCCCTACGAC, reverse CGGTGGGGTTGAGGATCT; Slug: forward TGTTTGCAAGATCTGCGGC, reverse TGCAGTCAGGGCAAGAAAAA; TIMP-1: GGCTTCTGGCATCCTGTTGTTG, reverse AAGGTGGTCTGGTTGACTTCTGG; TIMP-2: forward TGGAAACGACATTTATGGCAACCC, reverse CTCCAACGTCCAGCGAGACC; TGF-β1: forward GTGCGGCAGTGGTTGAGC; reverse GGTAGTGAACCCGTTGATGTCC; LOX: forward GGATACGGCACTGGCTACTT; REVERSEGACGCCTGGATGTAGTAGGG. Amplifications were performed in a Bioer LineGene 9600 thermal cycler (Bioer, Hangzhou, China). Gene expression levels relative to that of GAPDH were calculated using the ΔCT method.

### 2.3. Immunofluorescence

Confocal microscopy was used to assess the expression and localization of E-cadherin, N-cadherin, and vimentin, as well as actin cytoskeleton organization, in three independent experiments. Then, 2D monolayers of melanoma cells were cultured on rounded coverslips 12 mm in diameter while 3D spheroids were kept free-floating to be processed for the immunofluorescence procedures. After washing in phosphate-buffered saline (PBS), 2D monolayers and 3D spheroids were fixed in 4% paraformaldehyde in PBS-containing 2% sucrose for 10 and 30 min, respectively, at room temperature, post-fixed in 70% ethanol and stored at −20 °C until use. Samples were incubated with the primary antibodies: mouse anti-E-cadherin (1:2500, Becton Dickinson, Milan, Italy), rabbit anti-N-cadherin (1:200, Santa Cruz Biotechnology Inc., Dallas, TX, USA), and mouse anti-vimentin (1:200, Novocastra, Leica Microsystems, Milan, Italy). Alexa 488-conjugated secondary antibodies (1:500, Molecular Probes, Invitrogen, Monza, Italy) were incubated for 1 hr at room temperature in PBS. Samples which were incubated with the primary antibody omitted served as negative controls. The actin cytoskeleton was analyzed using 25 µM rhodamine-phalloidin (Sigma-Aldrich, Milan, Italy) added to the secondary antibody. Finally, nuclei were stained using 4′,6-diamidine-2′-phenylindole dihydrochloride (DAPI) (1:100,000, Sigma-Aldrich, Milan, Italy), the samples were mounted on glass slides using mowiol and observed using a laser scanning confocal microscope Leica TCS SP8 X (Leica Microsystems GmbH, Mannheim, Germany) equipped with the Leica LAS X rel. 3.1.1.15751 software (Leica Microsystems GmbH, Mannheim, Germany) to digitalize the images.

### 2.4. Western Blot

Whole cell lysates were obtained in RIPA buffer as previously described [37]. Briefly, samples were lysed on ice for 30 min and centrifuged at 14,000× *g* for 15 min at 4 °C to remove cell debris. Cell lysates (20 μg of total proteins), diluted in Sample buffer (Bio-Rad Laboratories, Segrate, MI, Italy), were run using polyacrylamide gel (SDS-PAGE) under reducing and denaturing conditions and transferred onto nitrocellulose membranes. Membranes were blocked and incubated with the primary antibodies: E-cadherin (1:2500, Becton Dickinson, Milan, Italy), N-cadherin (1:1000, Cell Signaling Technology Inc., Danvers, MA, USA), and vimentin (1:1000, Leica-Microsystems, Milan, Italy). Immunoreactive bands were detected after incubation with horseradish peroxidase-conjugated secondary antibodies (Cell Signaling Technology Inc.) and enhanced chemiluminescence Westar Eta C Ultra 2.0 reagents (Cyanagen, Bologna, Italy). Membranes were reprobed with α-tubulin (1:2000, Sigma-Aldrich, Milan, Italy) which was used for normalization. Each experiment was repeated three times.

### 2.5. SDS-Zymography

Serum-free cell culture media obtained from cells cultured in 2D monolayers and 3D spheroids (two independent copies of the same cell line grown in 2D or 3D) were mixed 3:1 with a sample buffer (containing 10% SDS). Samples (5 μg of total proteins) were run at 4 °C under non-reducing conditions on 10% SDS-PAGE co-polymerized with 1 mg/mL of type I gelatin. After electrophoresis, the gels were washed in 2.5% Triton X-100 (2 washes for 30 min each), and incubated overnight in an incubation buffer (Tris-HCl 50 mM, CaCl_2_ 5 mM, 0.02% NaN_3_, pH 7.5) at 37 °C. Gels were stained with Coomassie brilliant blue R250 to reveal MMPs’ gelatinolytic activity which appeared as clear bands on a blue background, and were quantified by densitometric analysis (UVBand, Eppendorf, Milan, Italy). Each experiment was repeated three times.

### 2.6. Statistical Analysis

Data were analyzed using GraphPad Prism v 9.3 software (GraphPad Software Inc., La Jolla, CA, USA). Data were expressed as mean ± standard deviation (SD). One-way ANOVA was used to compare the experimental groups. The *p* values lower than 0.05 were considered significant.

## 3. Results

### 3.1. Characterization of Melanoma Cell Morphology and EMT-Related Phenotype in 2D and 3D Cultures

**Figure 1:**
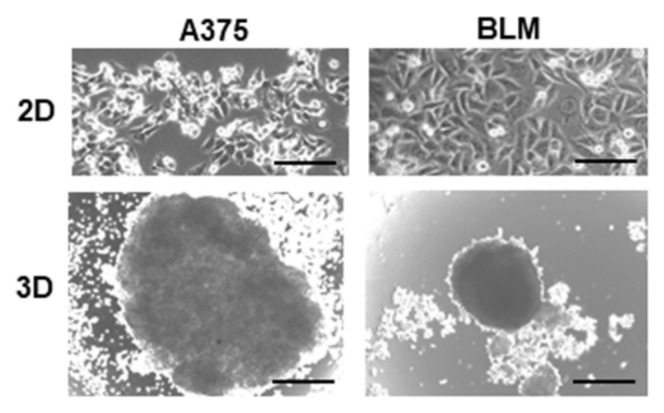
Representative micrographs from a phase contrast microscope showing cell morphology of A375 and BLM melanoma cells grown in 2D monolayers and 3D spheroids. While BLM cells formed spheroids containing densely packed cells, A375 cells formed less densely packed spheroids. Original magnification 10×. Scale bar: 200 µm.

According to the ATCC description (https://www.atcc.org/products/crl-1619, accessed on 20 October 2023), A375 cells derive from a primary melanoma of the skin and exhibit an epithelial phenotype, with cells having a polygonal shape and growing tightly apposed when cultured in 2D monolayers. The BLM cell line, obtained from lung metastases (https://www.cellosaurus.org/CVCL_7035, accessed on 20 October 2023), grew tightly apposed similarly to A375 cells when cultured in 2D monolayers. When cultured in 3D spheroids, A375 cells formed less compact and bigger 3D aggregates containing loosely apposed cells when compared to BLM cells. By contrast, BLM 3D spheroids were densely and tightly packed (Figure 1).

To characterize the EMT-related phenotype of A375 and BLM cells, the expression of adhering cell junctions, E-cadherin and N-cadherin, at the mRNA and protein level was investigated.

**Figure 2:**
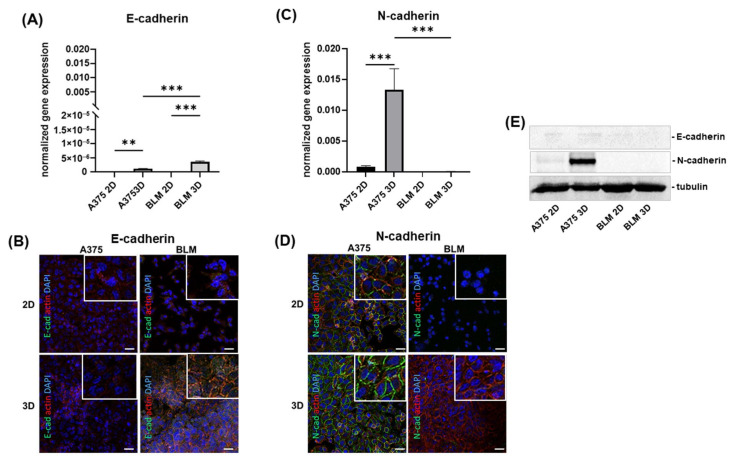
Bar graphs showing E-cadherin (**A**) and N-cadherin (**C**) mRNA levels in A375 and BLM melanoma cells. Data are expressed as means ± SD. Representative micrographs of confocal microscopy showing E-cadherin (**B**), N-cadherin (**D**), and actin cytoskeleton (**B**,**D**) in A375 and BLM cells cultured in 2D monolayers and 3D spheroids. The insets show the pattern of expression at higher magnification. The mesenchymal phenotype-related N-cadherin is expressed at cell boundaries only in A375 cells. However, cortical actin was evident in BLM 3D spheroids, suggesting that this epithelial feature is influenced by the 3D arrangement. Green: E-cadherin (**B**) and N-cadherin (**D**); red: actin; blue: DAPI. Original magnification: 40×. Scale bar: 25 µm. (**E**) Representative Western blot analysis of whole cell lysates showing E-cadherin and N-cadherin protein levels. Duplicate samples for each cell line and experimental condition were run. Each experiment was repeated three times. ** *p* < 0.01; *** *p* < 0.001.

E-cadherin mRNA was almost undetectable in A375 and BLM 2D monolayers. Albeit being expressed at very low levels, E-cadherin mRNA was significantly increased in their 3D counterparts and in BLM compared to A375 3D spheroids (*p* < 0.01 and *p* < 0.001, respectively) (Figure 2A). According to the gene expression analysis, confocal microscopy analysis confirmed that E-cadherin was expressed at a very low extent both in 2D and 3D cultures; however, few immunoreactive cells in BLM 3D spheroids were detectable. Notably, E-cadherin positivity remained at the cytoplasmic level and not at the cell boundary in the plasmalemma, indicating that the cell junctions are nonfunctional (Figure 2B).

A different N-cadherin gene expression pattern was observed, showing undetectable mRNA levels in BLM cells. However, a significant N-cadherin up-regulation was induced by 3D arrangement in A375 cells (*p* < 0.001) (Figure 2C). Confocal microscopy analysis confirmed the molecular data. N-cadherin immunoreactivity was more evident in A375 3D spheroids compared to 2D monolayers, while it was undetectable in BLM. In A375 cells, in both 2D and 3D cultures, N-cadherin was localized at the plasma membrane revealing that N-cadherin-containing cell junctions are functional in these cells (Figure 2D). Gene expression and confocal microscopy results were consistent with Western blot analysis for N-cadherin, while the E-cadherin immunoreactive bands were very faint and almost undetectable (Figure 2E).

Confocal microscopy analysis of actin cytoskeleton arrangement revealed that cortical actin, typical of epithelial cells, was detected in both cell lines. A low immunoreactivity was observed in A375 2D monolayers and 3D spheroids, and in BLM 2D monolayers as well, but it was strongly expressed in BLM 3D spheroids, indicating that both of these cell lines exhibit epithelial-related characteristics that become more evident in BLM cells when cultured in 3D (Figure 2B,D).

**Figure 3:**
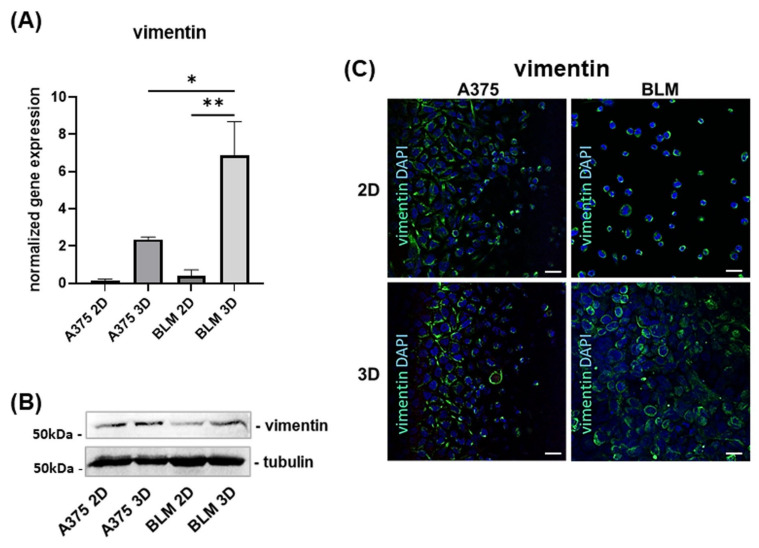
Bar graphs showing the mesenchymal marker vimentin mRNA levels in A375 and BLM melanoma cells (**A**). Data are expressed as means ± SD. (**B**) Representative Western blot analysis for vimentin expression in whole cell lysates showing its up-regulation in BLM 3D spheroids compared to 2D monolayers. (**C**) Representative micrographs of confocal microscopy showing vimentin expression in A375 and BLM cultured in different experimental settings. Green: vimentin; blue: DAPI. Original magnification: 40×. Scale bar: 25 µm. Duplicate samples for each cell line and experimental condition were run. Each experiment was repeated three times. * *p* < 0.05; ** *p* < 0.01.

Vimentin gene expression tended to increase in A375 (*p* ns) and BLM 3D spheroids (*p* < 0.01) compared to 2D monolayers. Moreover, vimentin gene expression was strongly and significantly increased in BLM compared to A375 3D spheroids (*p* < 0.05) (Figure 3A). By contrast, Western blot analysis revealed a non-significant and similar fold increase in vimentin protein levels in A375 and BLM 3D cells compared to their 2D monolayer counterparts. This expression level was similar in the two cell lines cultured in 3D (Figure 3B).

Vimentin confocal microscopy analysis, which showed immunoreactivity to be detectable in both A375 and BLM cells, and more evident in 3D spheroids, was consistent with Western blot data (Figure 3C).

### 3.2. Zeb1, Zeb2, Twist, Snail and e Slug Expression

**Figure 4:**
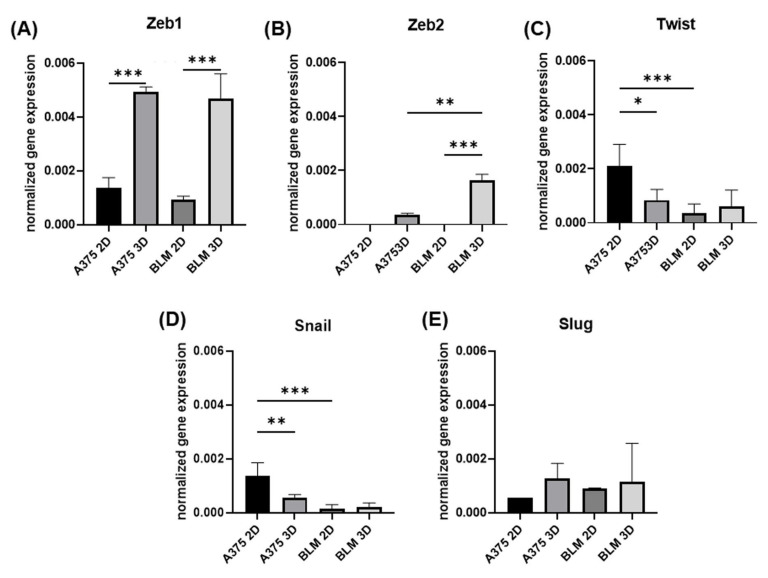
Bar graphs showing Zeb1 (**A**), Zeb2 (**B**), Twist (**C**), Snail (**D**) and Slug (**E**) gene expression in A375 and BLM melanoma cells grown in 2D monolayers and 3D spheroids assessed by real-time PCR. Data were normalized by GAPDH gene expression and are expressed as mean ± SD. Duplicate samples for each cell line and experimental condition were run. Each experiment was repeated three times. * *p* < 0.05; ** *p* < 0.01; *** *p* < 0.001.

Zeb1 mRNA levels were similarly expressed in A375 and BLM melanoma cells. In both cell lines, Zeb1 was strongly up-regulated in 3D spheroids compared to 2D monolayers (*p* < 0.001) (Figure 4A). Zeb 2 exhibited a similar pattern since it was undetectable in 2D monolayers but up-regulated in 3D spheroids (*p* ns and *p* < 0.01, respectively, for A375 and BLM). A significant up-regulation was evident in 3D BLM compared to 3D A375 (*p* < 0.005) (Figure 4B).

The Twist and Snail gene expressions were similar, revealing an opposite trend when compared to Zeb. They were expressed to higher extents in A375 compared to BLM cells (*p* < 0.001 for A375 2D vs. BLM 2D), and, although unaffected by 3D arrangement in BLM cells, the Snail mRNA levels were significantly lower in A375 3D spheroids compared to A375 2D monolayers (*p* < 0.01) (Figure 4C,D). No evident differences were detected for Slug gene expression (Figure 4E).

### 3.3. Invasive Potential

**Figure 5:**
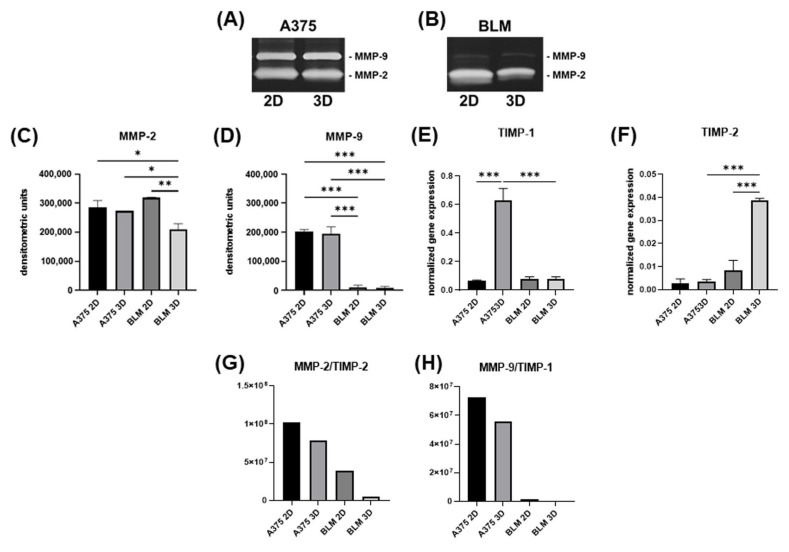
Representative SDS-zymography showing MMP-2 and MMP-9 activity assayed in serum-free cell supernatants of A375 (**A**) and BLM (**B**) cells. Bar graphs showing MMP-2 (**C**) and MMP-9 (**D**) activity after densitometric analysis of lytic bands. TIMP-1 (**E**) and TIMP-2 (**F**) gene expression analyzed by real-time PCR. Data are expressed as means ± SD. (**G**) MMP-2/TIMP-2 ratio and (**H**) MMP-9/TIMP-1 ratio are shown to predict the effective ECM degradation. Duplicate samples for each cell line and experimental condition were run. Each experiment was repeated three times. * *p* < 0.05, ** *p* < 0.01, *** *p* < 0.001.

The invasive potential of melanoma cells was assessed by analyzing MMP-2 and -9 activity in cell culture supernatants using SDS-zymography. Zymograms showed a different pattern of MMP levels in A375 and BLM cells. Indeed, A375 cells have high levels of both MMP-2 and MMP-9 (Figure 5A), while in BLM cells MMP-9 is almost undetectable (Figure 5B). A densitometric analysis of MMP-2 lytic bands revealed a similar activity of this gelatinase in A375 cells cultured in 2D monolayer and 3D spheroids. By contrast, MMP-2 activity was significantly decreased in BLM 3D spheroids compared to 2D monolayers (*p* < 0.01), as well as compared to A375 3D spheroids (*p* < 0.05) (Figure 5C). Different MMP-9 expression was also detectable in the two considered cell lines. Indeed, very evident and similar MMP-9 lytic bands were detected in 2D and 3D A375 cells. By contrast, MMP-9 was almost undetectable in BLM cell supernatants. Densitometric analysis showed a significant down-regulation of MMP-9 in 2D BLM compared to A375 2D monolayers (*p* < 0.001), as well as in BLM 3D spheroids compared to A375 3D spheroids (*p* < 0.001) (Figure 5D).

Gene expression for TIMP-1, the main inhibitor of MMP-9 [41], was significantly up-regulated in A375 spheroids compared to 2D monolayers (*p* < 0.001), but was expressed to a lower, but similar, extent in both 2D and 3D BLM cells (Figure 5E). A different pattern was detected for TIMP-2, the main inhibitor of MMP-2 [41], which was significantly up-regulated in 3D compared to 2D BLM cells (*p* < 0.001), while it remained expressed to a lower extent in both 2D and A375 cells (Figure 5F). Although the TIMP gene expression pattern was different in A375 and BLM cells, it was strongly affected by 3D arrangement in both of the cell lines. The MMP-2/TIMP-2, MMP-9, and TIMP-1 ratio was lower in BLM compared to A375 cells (Figure 5G and Figure 5H, respectively).

### 3.4. TGF-β1 and LOX Gene Expression

**Figure 6:**
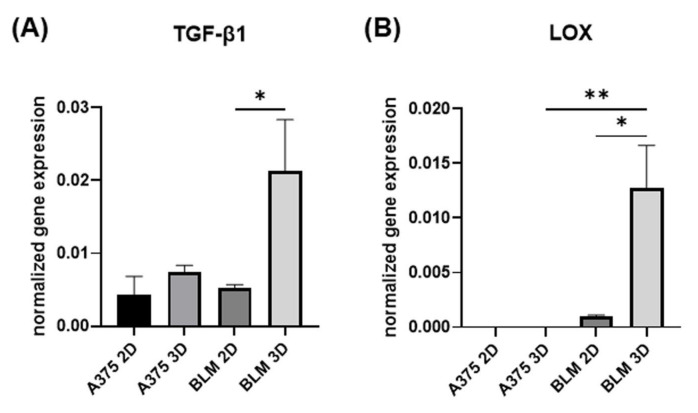
Bar graphs showing TGF-β1 (**A**) and LOX (**B**) mRNA levels in A375 and BLM cells analyzed by real-time PCR. Data are expressed as mean ± SD. Duplicate samples for each cell line and experimental condition were run. Each experiment was repeated three times. * *p* < 0.05, ** *p* < 0.01.

TGF-β1 is a master controller of EMT, able to influence the gene expression of EMT markers [5,8]. Its gene expression pattern was affected by the 3D arrangement, inducing a significant TGF-β1 up-regulation in 3D BLM compared to 2D monolayers. Conversely, TGF-β1 expression was similar in both 2D and 3D A375 cells. However, higher TGF-β1 mRNA levels were detected in 3D BLM compared to 3D A375 cells (Figure 6A).

Since LOX and the LOX family members (LOXL 1-4) are expressed and up-regulated in melanoma [28], we investigated LOX gene expression in our experimental models. LOX mRNA levels mirrored TGF-β1 gene expression and were significantly up-regulated in BLM 3D spheroids compared with 2D monolayers, while undetectable in A375 cells (Figure 6B).

**Figure 7:**
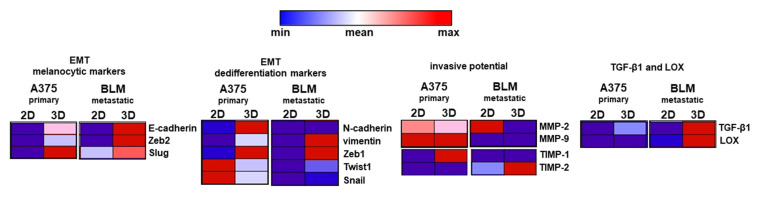
Heatmap summarizing the phenotypic profile of A375 and BLM cells cultured in 2D monolayers and 3D spheroids. The effect of 3D arrangement on cell phenotype related to the expression of the different considered markers was evident in both cell lines.

The heatmap, summarizing the overall results, shows that the two cell lines exhibited different EMT-related profiles, according to their different origins, and that the 3D arrangement impacted on EMT-related markers, as well as the invasive potential and the expression of TGF-β1 and LOX (Figure 7).

## 4. Discussion

Melanomagenesis is driven by a complex transcriptional program resembling those occurring during melanocyte development, and melanocytes undergo an EMT program characterized by the progressive acquisition of different phenotypic states [42,43] Interestingly, melanomagenesis is driven by a complex transcriptional program resembling those occurring during melanocyte development, characterized by substantial genetic variability [44,45]. To date, up to seven different melanoma cell phenotypes have been described [20,21,22,46,47], ranging from the proliferative (melanocytic) and the invasive/mesenchymal-like to the undifferentiated states [23].

In the present study, we aimed to characterize the morphological and molecular features of melanoma cell lines derived from a primary tumor (A375) or a metastatic lesion (BLM) in order to investigate their phenotype in relation to the expression of the main EMT markers, and whether these markers can be affected by the 3D arrangement.

Although melanoma cells are not of epithelial origin, they exhibit a spectrum of typical EMT markers and undergo an EMT-like process mirroring the classical EMT program observed in carcinomas [10]. This also includes the so-called “cadherin switch” [11], generally associated with a worse clinical stage and a poor prognosis in cancer patients [48], and the acquisition of a mesenchymal metastatic phenotype in cancer cells that leave the epidermal layer [49,50]. Indeed, N-cadherin expression was suggested to facilitate the escape of tumor cells from the control of resident keratinocytes in the epidermis [51,52,53], providing them an invasive behavior favored by the homophilic interactions with fibroblasts or endothelial cells in the tumor microenvironment [51,54], and also promoting cell survival and migration [55].

Herein, we show that E-cadherin gene expression was undetectable in A375 and BLM cell 2D monolayers, while very low levels were detected in 3D spheroids, with a significant up-regulation in BLM compared to A375 spheroids. Although the epithelial marker, E-cadherin was expressed at low levels, cortical actin, typical of epithelial cells, was detected in both A375 and BLM cells grown in 3D spheroids, with BLM revealing more intense immunoreactivity at the cell plasma membrane.

Conversely, mRNA for the mesenchymal marker N-cadherin was expressed only in A375 cells and up-regulated in 3D compared to 2D cell cultures. These findings are consistent with the reported E-cadherin down-regulation in melanoma tumor tissue compared with benign melanocytes and melanocytic nevi [56,57], suggesting a more mesenchymal/dedifferentiated phenotype for A375 cells that also retain some epithelial characteristics.

A high degree of heterogeneity of N- and E-cadherin expression was previously observed in melanoma cells, and a high percentage of cutaneous melanomas displayed a strong E-cadherin expression, even in advanced and invasive stages [58,59]. This is consistent with hybrid phenotypic states for melanoma cells [10,60].

In melanoma, elevated levels of vimentin, a mesenchymal EMT marker, are associated with the high invasive and migratory potential of cancer cells [61,62] and with a poor clinical outcome [63]. Our results show that vimentin is expressed in both A375 and BLM cells, but with a different pattern of expression. Indeed, it was up-regulated in 3D spheroids compared to 2D monolayers and was similarly expressed in A375 and BLM cells. Interestingly, the expression of vimentin was not paralleled by the expression of the mesenchymal marker N-cadherin, that was detected only in A375 cells.

These findings suggest that both A375 and BLM cells exhibit hybrid phenotypes, characterized by the concomitant expression of epithelial and mesenchymal markers but to different extents; more importantly, they indicate that 3D arrangement strongly affects their expression pattern.

Since the E-cadherin, cortical actin, N-cadherin, and vimentin expression are not sufficient to clearly define whether the hybrid phenotype is more melanocytic/differentiated or more dedifferentiated/mesenchymal, we also analyzed the expression of the main transcription factors driving the EMT process [8,13,14,24].

The EMT transcription factor Snail/SNAI1, undetectable in primary human melanocytes, plays a role during the phenotypic transition leading to malignant melanomas, and its expression is considered a characteristic of the undifferentiated/mesenchymal state of melanoma [8,10,64,65]. Our results show that Snail was significantly down-regulated in A375 spheroids compared to 2D monolayers, while it was similarly expressed in BLM cells in both experimental conditions, revealing very low Snail mRNA levels. Interestingly, Snail and E-cadherin expression was inversely related, as previously observed in bladder, colorectal, and pancreatic carcinomas [66,67]. In our experimental setting, Snail gene expression was mirrored by Twist1 mRNA levels, which were significantly down-regulated in A375 3D spheroids. The inverse relationship between Snail and vimentin gene expression was evident in A375 cells, supporting the hypothesis of a hybrid/intermediate phenotype for this cell line.

While Slug/SNAI2 promotes a mesenchymal state in epithelial tumors, it is a melanocytic marker [68], but it also acts as an activator of Zeb1 transcription, leading to the downregulation of E-cadherin [69]. Therefore, the role of Slug in melanoma progression still needs to be clarified [70]. Here, we did not observe significant differences in Slug expression in A375 or BLM cells in either experimental setting. However, an inverse relationship between Slug and Snail expression was evident in A375 cells, confirming their divergent roles in melanoma cells.

Zeb1 and Zeb2, both mesenchymal markers in the epithelial context, are inversely related in melanoma [10]. Indeed, similarly to carcinomas, Zeb1 is a repressor of E-cadherin expression and promotes dedifferentiation [71,72]. A high expression in tumor tissues of Zeb1, and also of Twist, was found to positively correlate with tumor progression and a significantly reduced metastasis-free survival [72]. By contrast, Zeb2 is a marker of melanocytic differentiation and, interestingly, does not promote invasion [73,74]. In line with these observations, our findings revealed higher Zeb1 compared to Zeb2 mRNA levels in A375 and BLM cells, which are both cancer cells, and demonstrated that these transcription factors were both up-regulated by a 3D arrangement. Interestingly, Zeb2 was significantly increased in 3D BLM compared to 3D A375 cells, and 3D arrangement significantly affected Zeb2 expression only in BLM cells. Considering the role of Zeb2 as a melanocytic differentiation marker, this finding leads to the hypothesis that BLM cells are more “melanocytic” than A375 cells. This hypothesis is consistent with the EMT markers, which suggested for A375 a more mesenchymal/undifferentiated phenotype.

Cells undergoing EMT are characterized by an increased invasive potential based on MMP-2 and MMP-9 activity [75], leading to distant metastases [76,77] and the formation of microvascular channels [78]. High levels of MMP-2 were associated with poor prognosis in melanoma patients [79], as well as in BLM xenografts with a significant correlation with the increased malignancy [80]. Our results show high MMP-2 activity in melanoma cells, as previously described [80], that was not affected by the 3D arrangement in A375 cells. By contrast, a significant down-regulation was evident in BLM 3D spheroids compared to 2D monolayers but also compared to A375 3D spheroids, indicating the more invasive potential of A375 cells. This hypothesis is also supported by the significantly higher MMP-9 activity in A375 compared to BLM cell supernatants, both in 2D monolayers and 3D spheroids. Indeed, high levels of MMP-9 have been described in melanoma patients and its expression has been suggested to be a tumor progression marker [77]. Although the role of MMP-9 in melanoma is not completely clear, our results which show a lower level of MMP-9 activity in BLM cells obtained from melanoma lung metastases, are consistent with previous observations of its higher expression in human melanoma cells in the radial growth phase of primary melanoma, suggesting a role in early phases of melanoma invasion [81]. Interestingly, MMP-9 could exert proangiogenic activity by favoring the release of matrix-sequestered angiogenic factors in the early stages of tumor development [82].

To better speculate about A375 and BLM invasive potential, the MMPs/TIMPs balance is predictive of the overall MMPs activity and cell behavior [83]. We found that A375 and BLM cells also differ in the opposite patterns of TIMP-1 and TIMP-2, also differently affected by the 3D arrangement, with a significant up-regulation of TIMP-1 in A375 cells and of TIMP-2 in BLM. This pattern, considered in relation to MMP-2 and -9 activity, may suggest that TIMP-1 expression could likely strengthen the lower gelatinolytic activity in BLM cells. By contrast, these gelatinases could be strongly inhibited by TIMP-2 in both A375 and BLM 3D spheroids, and the MMP-2/TIMP-2 balance likely suggests a lower invasive potential in the metastatic BLM cell line. In this complex scenario, confirming the different phenotypes of A375 and BLM cells in relation to their invasive potential, is important to consider that TIMPs not only inhibit MMPs activity, but also are involved in cancer cell proliferation and tumor growth [84]. Moreover, TIMP-2 exerts a dual and opposite regulation on MMP-2 since it acts as key player in MMP-2 activation by MT-MMP-1 [85,86].

TGF-β1 acts as a master controller of EMT, thus promoting the distinctive EMT-related phenotypic changes [12,13,24,74,87], but it is also a promotor of melanoma progression independently of EMT [88], also favoring the immune escape of melanoma cells in vitro and in vivo [89]. As a consequence, TGF-β gene expression could be unrelated to the expression of EMT markers in melanoma cells, as observed in our experimental setting. According to this suggestion, we observed a TGF-β1 up-regulation in BLM cells, which were metastatic, grown in 3D spheroids.

LOX and the LOX family members (LOXL 1-4) are up-regulated in several melanoma cell lines while their down-regulation or inhibition reduces the invasion of melanoma cells [28,90]. While undetectable in melanocytes and primary melanoma cells, LOX up-regulation was described in metastatic BLM cells and in the vertical growth phase of the WM793 melanoma cell line [28]. In our study, we investigated the LOX gene expression in relation to the EMT-related phenotype and differentiation state of melanoma cells for the first time. Our results confirm that the highest gene expression of LOX was found in the metastatic and less dedifferentiated BLM cells that were isolated from lung metastases compared to A375 cells, which revealed an almost undetectable expression. Interestingly, LOX was strongly affected by the 3D arrangement, which induced a significant up-regulation in BLM 3D spheroids compared to BLM 2D monolayer and A375 3D spheroids as well. Moreover, the up-regulation of LOX in BLM 3D spheroids was paralleled by TGF-β1 gene expression, which was able to induce LOX and LOX family gene expression [91].

## 5. Conclusions

**Figure 8:**
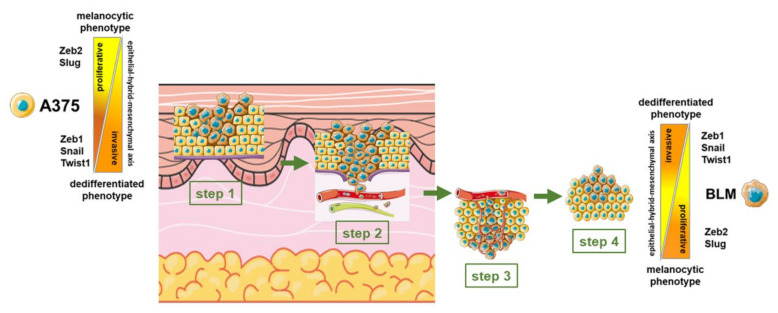
Diagram summarizing the different phases of melanoma progression and the phenotypic states of melanoma cells ranging from melanocytic to dedifferentiated phenotypes [10,24]. In the early phases, during the radial growth of melanoma, cells remain in the epidermal layer (1). With vertical growth, melanoma cells invade the connective tissue (2). Finally, melanoma cells are disseminated through the blood and lymphatic circulation (3) to form distant metastases (4). In this vertical gradient, from the upper to the deeper part of invasive melanoma, an epithelial—hybrid—mesenchymal axis can be described, characterized by up to seven phenotypic states of melanoma cells. In the intermediate/hybrid states, both epithelial and mesenchymal markers can be detected in melanoma cells. Our results lead to the hypothesis that A375 cells, isolated from a primary tumor, are characterized by a more dedifferentiated phenotype to favor tumor invasion in the early phases of progression (step 1). By contrast, BLM cells, which were isolated from a lung metastasis, have a more epithelial/differentiated phenotype to favor colonization of the metastases (steps 3–4).

Overall, our results show that A375 and BLM melanoma cells grown in 2D monolayers and 3D spheroids exhibit a differential expression of EMT-related markers, which is strongly affected by the 3D arrangement. Our results show that both of these cells displayed intermediate/hybrid phenotypes with the concomitant expression of both epithelial and mesenchymal characteristics. However, A375 cells, which were highly malignant cells derived from a primary melanoma, exhibited a less epithelial/more mesenchymal phenotype that could favor the progression of melanoma in the early phases, requiring a more dedifferentiated phenotype; by contrast, BLM cells, which were obtained from lung metastases, had more melanocytic/fewer mesenchymal characteristics, which is consistent with growth of the metastasis at a distance (Figure 8). These phenotypic differences, together with the different signaling pathways activated by the BRAF and NRAS mutations, could be responsible for the different behavior of A375 and BLM cells.

These findings contribute new knowledge of the phenotypes of two different melanoma cell lines and support the importance of studying the characteristics and behavior of melanoma cells in 3D spheroids, which might represent an in vitro pre-clinical model able to identify tumor markers and study new therapeutic tools in melanoma, as previously demonstrated in other tumors [36,38,92,93].

## Data Availability

The data presented in this study are available on request from the corresponding author.

## References

[1] Siegel R.L., Miller K.D., Wagle N.S., Jemal A. (2023). Cancer statistics, 2023. CA Cancer J. Clin..

[2] Carr S., Smith C., Wernberg J. (2020). Epidemiology and Risk Factors of Melanoma. Surg. Clin. N. Am..

[3] Tímár J., Ladányi A. (2022). Molecular Pathology of Skin Melanoma: Epidemiology, Differential Diagnostics, Prognosis and Therapy Prediction. Int. J. Mol. Sci..

[4] Timis T., Bergthorsson J.T., Greiff V., Cenariu M., Cenariu D. (2023). Pathology and Molecular Biology of Melanoma. Curr. Issues Mol. Biol..

[5] Thiery J.P., Acloque H., Huang R.Y.J., Nieto M.A. (2009). Epithelial-mesenchymal transitions in development and disease. Cell.

[6] Huang Y., Hong W., Wei X. (2022). The molecular mechanisms and therapeutic strategies of EMT in tumor progression and metastasis. J. Hematol. Oncol..

[7] Zeisberg M., Neilson E.G. (2009). Biomarkers for epithelial-mesenchymal transitions. J. Clin. Investig..

[8] Kalluri R., Weinberg R.A. (2009). The basics of epithelial-mesenchymal transition. J. Clin. Investig..

[9] Lamouille S., Xu J., Derynck R. (2014). Molecular mechanisms of epithelial-mesenchymal transition. Nat. Rev. Mol. Cell Biol..

[10] Pedri D., Karras P., Landeloos E., Marine J.-C., Rambow F. (2022). Epithelial-to-mesenchymal-like transition events in melanoma. FEBS J..

[11] Alonso S.R., Tracey L., Ortiz P., Pérez-Gómez B., Palacios J., Pollán M., Linares J., Serrano S., Sáez-Castillo A.I., Sánchez L. (2007). A high-throughput study in melanoma identifies epithelial-mesenchymal transition as a major determinant of metastasis. Cancer Res..

[12] Pearlman R.L., Montes de Oca M.K., Pal H.C., Afaq F. (2017). Potential therapeutic targets of epithelial-mesenchymal transition in melanoma. Cancer Lett..

[13] Hodorogea A., Calinescu A., Antohe M., Balaban M., Nedelcu R.I., Turcu G., Ion D.A., Badarau I.A., Popescu C.M., Popescu R. (2019). Epithelial-Mesenchymal Transition in Skin Cancers: A Review. Anal. Cell. Pathol..

[14] Vandyck H.H., Hillen L.M., Bosisio F.M., van den Oord J., zur Hausen A., Winnepenninckx V. (2021). Rethinking the biology of metastatic melanoma: A holistic approach. Cancer Metastasis Rev..

[15] Nieto M.A., Cano A. (2012). The epithelial–mesenchymal transition under control: Global programs to regulate epithelial plasticity. Semin. Cancer Biol..

[16] Puisieux A., Brabletz T., Caramel J. (2014). Oncogenic roles of EMT-inducing transcription factors. Nat. Cell Biol..

[17] Chuang K.-T., Chiou S.-S., Hsu S.-H. (2023). Recent Advances in Transcription Factors Biomarkers and Targeted Therapies Focusing on Epithelial-Mesenchymal Transition. Cancers.

[18] Ozdamar B., Bose R., Barrios-Rodiles M., Wang H.-R., Zhang Y., Wrana J.L. (2005). Regulation of the polarity protein Par6 by TGFbeta receptors controls epithelial cell plasticity. Science.

[19] Papageorgis P. (2015). TGFβ Signaling in Tumor Initiation, Epithelial-to-Mesenchymal Transition, and Metastasis. J. Oncol..

[20] Tirosh I., Izar B., Prakadan S.M., Wadsworth M.H., Treacy D., Trombetta J.J., Rotem A., Rodman C., Lian C., Murphy G. (2016). Dissecting the multicellular ecosystem of metastatic melanoma by single-cell RNA-seq. Science.

[21] Chapman A., Del Fernandez Ama L., Ferguson J., Kamarashev J., Wellbrock C., Hurlstone A. (2014). Heterogeneous tumor subpopulations cooperate to drive invasion. Cell Rep..

[22] Hoek K.S., Eichhoff O.M., Schlegel N.C., Döbbeling U., Kobert N., Schaerer L., Hemmi S., Dummer R. (2008). In vivo switching of human melanoma cells between proliferative and invasive states. Cancer Res..

[23] Rambow F., Marine J.-C., Goding C.R. (2019). Melanoma plasticity and phenotypic diversity: Therapeutic barriers and opportunities. Genes Dev..

[24] Tang Y., Durand S., Dalle S., Caramel J. (2020). EMT-Inducing Transcription Factors, Drivers of Melanoma Phenotype Switching, and Resistance to Treatment. Cancers.

[25] Ranjan A., Kalraiya R.D. (2014). Invasive potential of melanoma cells correlates with the expression of MT1-MMP and regulated by modulating its association with motility receptors via N-glycosylation on the receptors. Biomed. Res. Int..

[26] El Kharbili M., Cario M., Béchetoille N., Pain C., Boucheix C., Degoul F., Masse I., Berthier-Vergnes O. (2020). Tspan8 Drives Melanoma Dermal Invasion by Promoting ProMMP-9 Activation and Basement Membrane Proteolysis in a Keratinocyte-Dependent Manner. Cancers.

[27] Napoli S., Scuderi C., Gattuso G., Di Bella V., Candido S., Basile M.S., Libra M., Falzone L. (2020). Functional Roles of Matrix Metalloproteinases and Their Inhibitors in Melanoma. Cells.

[28] Kielosto M., Eriksson J., Nummela P., Yin M., Hölttä E. (2018). Divergent roles of lysyl oxidase family members in ornithine decarboxylase- and RAS-transformed mouse fibroblasts and human melanoma cells. Oncotarget.

[29] Vázquez-Naharro A., Bustos-Tauler J., Floristán A., Yuste L., Oltra S.S., Vinyals A., Moreno-Bueno G., Fabra À., Portillo F., Cano A. (2022). Loxl3 Promotes Melanoma Progression and Dissemination Influencing Cell Plasticity and Survival. Cancers.

[30] Wang W., Wang X., Yao F., Huang C. (2022). Lysyl Oxidase Family Proteins: Prospective Therapeutic Targets in Cancer. Int. J. Mol. Sci..

[31] Fontana F., Marzagalli M., Sommariva M., Gagliano N., Limonta P. (2021). In Vitro 3D Cultures to Model the Tumor Microenvironment. Cancers.

[32] El Harane S., Zidi B., El Harane N., Krause K.-H., Matthes T., Preynat-Seauve O. (2023). Cancer Spheroids and Organoids as Novel Tools for Research and Therapy: State of the Art and Challenges to Guide Precision Medicine. Cells.

[33] Manduca N., Maccafeo E., de Maria R., Sistigu A., Musella M. (2023). 3D cancer models: One step closer to in vitro human studies. Front. Immunol..

[34] Ohguro H., Watanabe M., Sato T., Hikage F., Furuhashi M., Okura M., Hida T., Uhara H. (2023). 3D Spheroid Configurations Are Possible Indictors for Evaluating the Pathophysiology of Melanoma Cell Lines. Cells.

[35] Gagliano N., Sforza C., Sommariva M., Menon A., Conte V., Sartori P., Procacci P. (2017). 3D-spheroids: What can they tell us about pancreatic ductal adenocarcinoma cell phenotype?. Exp. Cell Res..

[36] Gagliano N., Celesti G., Tacchini L., Pluchino S., Sforza C., Rasile M., Valerio V., Laghi L., Conte V., Procacci P. (2016). Epithelial-to-mesenchymal transition in pancreatic ductal adenocarcinoma: Characterization in a 3D-cell culture model. World J. Gastroenterol..

[37] Fontana F., Raimondi M., Marzagalli M., Sommariva M., Limonta P., Gagliano N. (2019). Epithelial-To-Mesenchymal Transition Markers and CD44 Isoforms Are Differently Expressed in 2D and 3D Cell Cultures of Prostate Cancer Cells. Cells.

[38] Fontana F., Raimondi M., Marzagalli M., Sommariva M., Gagliano N., Limonta P. (2020). Three-Dimensional Cell Cultures as an In Vitro Tool for Prostate Cancer Modeling and Drug Discovery. Int. J. Mol. Sci..

[39] Kutle I., Polten R., Hachenberg J., Klapdor R., Morgan M., Schambach A. (2023). Tumor Organoid and Spheroid Models for Cervical Cancer. Cancers.

[40] Fedorenko I.V., Gibney G.T., Smalley K.S.M. (2013). NRAS mutant melanoma: Biological behavior and future strategies for therapeutic management. Oncogene.

[41] Jackson H.W., Defamie V., Waterhouse P., Khokha R. (2017). TIMPs: Versatile extracellular regulators in cancer. Nat. Rev. Cancer.

[42] Centeno P.P., Pavet V., Marais R. (2023). The journey from melanocytes to melanoma. Nat. Rev. Cancer.

[43] Belote R.L., Le D., Maynard A., Lang U.E., Sinclair A., Lohman B.K., Planells-Palop V., Baskin L., Tward A.D., Darmanis S. (2021). Human melanocyte development and melanoma dedifferentiation at single-cell resolution. Nat. Cell Biol..

[44] Shain A.H., Yeh I., Kovalyshyn I., Sriharan A., Talevich E., Gagnon A., Dummer R., North J., Pincus L., Ruben B. (2015). The Genetic Evolution of Melanoma from Precursor Lesions. N. Engl. J. Med..

[45] Sanna A., Harbst K., Johansson I., Christensen G., Lauss M., Mitra S., Rosengren F., Häkkinen J., Vallon-Christersson J., Olsson H. (2020). Tumor genetic heterogeneity analysis of chronic sun-damaged melanoma. Pigment Cell Melanoma Res..

[46] Karras P., Bordeu I., Pozniak J., Nowosad A., Pazzi C., van Raemdonck N., Landeloos E., van Herck Y., Pedri D., Bervoets G. (2022). A cellular hierarchy in melanoma uncouples growth and metastasis. Nature.

[47] Ennen M., Keime C., Gambi G., Kieny A., Coassolo S., Thibault-Carpentier C., Margerin-Schaller F., Davidson G., Vagne C., Lipsker D. (2017). MITF-High and MITF-Low Cells and a Novel Subpopulation Expressing Genes of Both Cell States Contribute to Intra- and Intertumoral Heterogeneity of Primary Melanoma. Clin. Cancer Res..

[48] Krengel S., Grotelüschen F., Bartsch S., Tronnier M. (2004). Cadherin expression pattern in melanocytic tumors more likely depends on the melanocyte environment than on tumor cell progression. J. Cutan. Pathol..

[49] Murtas D., Maxia C., Diana A., Pilloni L., Corda C., Minerba L., Tomei S., Piras F., Ferreli C., Perra M.T. (2017). Role of epithelial-mesenchymal transition involved molecules in the progression of cutaneous melanoma. Histochem. Cell Biol..

[50] Loh C.-Y., Chai J.Y., Tang T.F., Wong W.F., Sethi G., Shanmugam M.K., Chong P.P., Looi C.Y. (2019). The E-Cadherin and N-Cadherin Switch in Epithelial-to-Mesenchymal Transition: Signaling, Therapeutic Implications, and Challenges. Cells.

[51] Hsu M., Andl T., Li G., Meinkoth J.L., Herlyn M. (2000). Cadherin repertoire determines partner-specific gap junctional communication during melanoma progression. J. Cell Sci..

[52] Lade-Keller J., Riber-Hansen R., Guldberg P., Schmidt H., Hamilton-Dutoit S.J., Steiniche T. (2013). E- to N-cadherin switch in melanoma is associated with decreased expression of phosphatase and tensin homolog and cancer progression. Br. J. Dermatol..

[53] Li G., Schaider H., Satyamoorthy K., Hanakawa Y., Hashimoto K., Herlyn M. (2001). Downregulation of E-cadherin and Desmoglein 1 by autocrine hepatocyte growth factor during melanoma development. Oncogene.

[54] Qi J., Chen N., Wang J., Siu C.-H. (2005). Transendothelial migration of melanoma cells involves N-cadherin-mediated adhesion and activation of the beta-catenin signaling pathway. Mol. Biol. Cell.

[55] Nguyen T., Mège R.M. (2016). N-Cadherin and Fibroblast Growth Factor Receptors crosstalk in the control of developmental and cancer cell migrations. Eur. J. Cell Biol..

[56] Silye R., Karayiannakis A.J., Syrigos K.N., Poole S., van Noorden S., Batchelor W., Regele H., Sega W., Boesmueller H., Krausz T. (1998). E-cadherin/catenin complex in benign and malignant melanocytic lesions. J. Pathol..

[57] Sanders D.S., Blessing K., Hassan G.A., Bruton R., Marsden J.R., Jankowski J. (1999). Alterations in cadherin and catenin expression during the biological progression of melanocytic tumours. Mol. Pathol..

[58] Danen E.H., de Vries T.J., Morandini R., Ghanem G.G., Ruiter D.J., van Muijen G.N. (1996). E-cadherin expression in human melanoma. Melanoma Res..

[59] Kim J.E., Leung E., Baguley B.C., Finlay G.J. (2013). Heterogeneity of expression of epithelial-mesenchymal transition markers in melanocytes and melanoma cell lines. Front. Genet..

[60] Cook D.P., Vanderhyden B.C. (2020). Context specificity of the EMT transcriptional response. Nat. Commun..

[61] Hendrix M.J., Seftor E.A., Chu Y.W., Seftor R.E., Nagle R.B., McDaniel K.M., Leong S.P., Yohem K.H., Leibovitz A.M., Meyskens F.L. (1992). Coexpression of vimentin and keratins by human melanoma tumor cells: Correlation with invasive and metastatic potential. J. Natl. Cancer Inst..

[62] Chu Y.W., Seftor E.A., Romer L.H., Hendrix M.J. (1996). Experimental coexpression of vimentin and keratin intermediate filaments in human melanoma cells augments motility. Am. J. Pathol..

[63] Li M., Zhang B., Sun B., Wang X., Ban X., Sun T., Liu Z., Zhao X. (2010). A novel function for vimentin: The potential biomarker for predicting melanoma hematogenous metastasis. J. Exp. Clin. Cancer Res..

[64] Brabletz T., Kalluri R., Nieto M.A., Weinberg R.A. (2018). EMT in cancer. Nat. Rev. Cancer.

[65] Poser I., Domínguez D., de Herreros A.G., Varnai A., Buettner R., Bosserhoff A.K. (2001). Loss of E-cadherin expression in melanoma cells involves up-regulation of the transcriptional repressor Snail. J. Biol. Chem..

[66] Batlle E., Sancho E., Francí C., Domínguez D., Monfar M., Baulida J., García De Herreros A. (2000). The transcription factor snail is a repressor of E-cadherin gene expression in epithelial tumour cells. Nat. Cell Biol..

[67] Cano A., Pérez-Moreno M.A., Rodrigo I., Locascio A., Blanco M.J., Del Barrio M.G., Portillo F., Nieto M.A. (2000). The transcription factor snail controls epithelial-mesenchymal transitions by repressing E-cadherin expression. Nat. Cell Biol..

[68] Shirley S.H., Greene V.R., Duncan L.M., Torres Cabala C.A., Grimm E.A., Kusewitt D.F. (2012). Slug expression during melanoma progression. Am. J. Pathol..

[69] Wels C., Joshi S., Koefinger P., Bergler H., Schaider H. (2011). Transcriptional activation of ZEB1 by Slug leads to cooperative regulation of the epithelial-mesenchymal transition-like phenotype in melanoma. J. Investig. Dermatol..

[70] Iwakami Y., Yokoyama S., Watanabe K., Hayakawa Y. (2018). STAM-binding protein regulates melanoma metastasis through SLUG stabilization. Biochem. Biophys. Res. Commun..

[71] Vandamme N., Berx G. (2014). Melanoma cells revive an embryonic transcriptional network to dictate phenotypic heterogeneity. Front. Oncol..

[72] Caramel J., Papadogeorgakis E., Hill L., Browne G.J., Richard G., Wierinckx A., Saldanha G., Osborne J., Hutchinson P., Tse G. (2013). A switch in the expression of embryonic EMT-inducers drives the development of malignant melanoma. Cancer Cell.

[73] Denecker G., Vandamme N., Akay O., Koludrovic D., Taminau J., Lemeire K., Gheldof A., de Craene B., van Gele M., Brochez L. (2014). Identification of a ZEB2-MITF-ZEB1 transcriptional network that controls melanogenesis and melanoma progression. Cell Death Differ..

[74] Vandamme N., Denecker G., Bruneel K., Blancke G., Akay Ö., Taminau J., de Coninck J., de Smedt E., Skrypek N., van Loocke W. (2020). The EMT Transcription Factor ZEB2 Promotes Proliferation of Primary and Metastatic Melanoma While Suppressing an Invasive, Mesenchymal-Like Phenotype. Cancer Res..

[75] Cheng Q., Wu J., Zhang Y., Liu X., Xu N., Zuo F., Xu J. (2017). SOX4 promotes melanoma cell migration and invasion though the activation of the NF-κB signaling pathway. Int. J. Mol. Med..

[76] Leonardi G.C., Falzone L., Salemi R., Zanghì A., Spandidos D.A., Mccubrey J.A., Candido S., Libra M. (2018). Cutaneous melanoma: From pathogenesis to therapy (Review). Int. J. Oncol..

[77] Guarneri C., Bevelacqua V., Polesel J., Falzone L., Cannavò P.S., Spandidos D.A., Malaponte G., Libra M. (2017). NF-κB inhibition is associated with OPN/MMP-9 downregulation in cutaneous melanoma. Oncol. Rep..

[78] Liang X., Sun R., Zhao X., Zhang Y., Gu Q., Dong X., Zhang D., Sun J., Sun B. (2017). Rictor regulates the vasculogenic mimicry of melanoma via the AKT-MMP-2/9 pathway. J. Cell. Mol. Med..

[79] Väisänen A., Kallioinen M., Taskinen P.J., Turpeenniemi-Hujanen T. (1998). Prognostic value of MMP-2 immunoreactive protein (72 kD type IV collagenase) in primary skin melanoma. J. Pathol..

[80] Hofmann U.B., Westphal J.R., van Muijen G.N., Ruiter D.J. (2000). Matrix metalloproteinases in human melanoma. J. Investig. Dermatol..

[81] van den Oord J.J., Paemen L., Opdenakker G., de Wolf-Peeters C. (1997). Expression of gelatinase B and the extracellular matrix metalloproteinase inducer EMMPRIN in benign and malignant pigment cell lesions of the skin. Am. J. Pathol..

[82] Bergers G., Brekken R., McMahon G., Vu T.H., Itoh T., Tamaki K., Tanzawa K., Thorpe P., Itohara S., Werb Z. (2000). Matrix metalloproteinase-9 triggers the angiogenic switch during carcinogenesis. Nat. Cell Biol..

[83] Gomez D.E., Alonso D.F., Yoshiji H., Thorgeirsson U.P. (1997). Tissue inhibitors of metalloproteinases: Structure, regulation and biological functions. Eur. J. Cell Biol..

[84] Sun J., Stetler-Stevenson W.G. (2009). Overexpression of tissue inhibitors of metalloproteinase 2 up-regulates NF-kappaB activity in melanoma cells. J. Mol. Signal..

[85] Strongin A.Y., Collier I., Bannikov G., Marmer B.L., Grant G.A., Goldberg G.I. (1995). Mechanism of cell surface activation of 72-kDa type IV collagenase. Isolation of the activated form of the membrane metalloprotease. J. Biol. Chem..

[86] Butler G.S., Butler M.J., Atkinson S.J., Will H., Tamura T., van Schade Westrum S., Crabbe T., Clements J., d’Ortho M.P., Murphy G. (1998). The TIMP2 membrane type 1 metalloproteinase “receptor” regulates the concentration and efficient activation of progelatinase A. A kinetic study. J. Biol. Chem..

[87] Liu Y. (2004). Epithelial to mesenchymal transition in renal fibrogenesis: Pathologic significance, molecular mechanism, and therapeutic intervention. J. Am. Soc. Nephrol..

[88] Cantelli G., Orgaz J.L., Rodriguez-Hernandez I., Karagiannis P., Maiques O., Matias-Guiu X., Nestle F.O., Marti R.M., Karagiannis S.N., Sanz-Moreno V. (2015). TGF-β-Induced Transcription Sustains Amoeboid Melanoma Migration and Dissemination. Curr. Biol..

[89] Li Z., Wang F., Dang J., Cheng F., Zheng F. (2022). Bidirectional regulation between tumor cell-intrinsic PD-L1 and TGF-β1 in epithelial-to-mesenchymal transition in melanoma. Transl. Cancer Res..

[90] Da Silva R., Uno M., Marie S.K.N., Oba-Shinjo S.M. (2015). LOX expression and functional analysis in astrocytomas and impact of IDH1 mutation. PLoS ONE.

[91] Sethi A., Mao W., Wordinger R.J., Clark A.F. (2011). Transforming growth factor-beta induces extracellular matrix protein cross-linking lysyl oxidase (LOX) genes in human trabecular meshwork cells. Investig. Ophthalmol. Vis. Sci..

[92] Eritja N., Dolcet X., Matias-Guiu X. (2013). Three-dimensional epithelial cultures: A tool to model cancer development and progression. Histol. Histopathol..

[93] Kunz-Schughart L.A. (1999). Multicellular tumor spheroids: Intermediates between monolayer culture and in vivo tumor. Cell Biol. Int..

